# Supplementary material for: How reliable is the use of ovarian dilatations for mosquito age-grading? A blinded multi-rater validation of the Polovodova method in Anopheles mosquitoes
Source: J Med Entomol. 2026 Oct 3;63(5):tjag157. doi: 10.1093/jme/tjag157 (PMC13633664; doi:10.1093/jme/tjag157)
Supplement: Supplementary file 1 [file tjag157_supplementary_data.docx]

# Supplementary Material

**Figure S1. Establishment of parity ‘reference group’**. Flow diagram illustrating the true classification of parous and nulliparous mosquitoes based on observations on blood-feeding history and oviposition outcome (based on R0), and gravid status (based on dissections made by R1-R3). All gravid mosquitoes were confirmed and classified as gravid only when fully developed eggs at Christophers stage V were confirmed present in the ovaries. Mosquitoes were classified as nulliparous, parous, or gravid. Individuals lacking clear parity determination were excluded.

**Figure S2**. **Establishment of assumed gonotrophic cycles.** Schematic showing the decision framework used to infer assumed gonotrophic cycle (AR) categories based on blood-feeding history, oviposition outcome, and gravid status. Mosquitoes were classified as nulliparous, parous (GC1-GC4), or gravid using biologically informed rules applied iteratively across successive blood meals. Gravid status was assigned only when fully developed eggs corresponding to Christophers stage V were confirmed in the ovaries.

**Table S1.** Inter-rater Agreement between reference data/ground truth (R0) and each of the other rates (R1, R2, R3)

| Species | Researcher 0 | | Researcher 1 | | IRR-*Kappa* | P-Value |
| --- | --- | --- | --- | --- | --- | --- |
|  | Nulliparous  n/N (prop) | Parous  n/N (prop) | Nulliparous  n/N (prop) | Parous  n/N (prop) |  |  |
| *An. arabiensis* | 439/472 (0.93) | 1254/1332 (0.94) | 439/517 (0.85) | 1254/1263 (0.99) | 0.87 [0.83, 0.92] | <0.001 |
|  |  |  | Researcher 2 | |  |  |
|  | 438/472 (0.93) | 1262/1332 (0.95) | 438/508 (0.86) | 1262/1272 (0.99) | 0.88 [0.83, 0.92] | <0.001 |
|  |  |  | Researcher 3 | |  |  |
|  | 440/472  (0.93) | 1265/1332  (0.95) | 440/507  (0.87) | 1265/1273  (0.99) | 0.89 [0.84, 0.93] | <0.001 |
|  |  |  |  |  |  |  |
|  | Researcher 0 | | Researcher 1 | |  |  |
| *An. funestus* | 362/365 (0.99) | 1198/1279 (0.94) | 362/442 (0.82) | 1198/1199 (1) | 0.86 [0.81, 0.91] | < 0.001 |
|  |  |  | Researcher 2 | |  |  |
|  | 362/365  (0.99) | 1217/1279  (0.95) | 362/423  (0.86) | 1217/1218 (1) | 0.89 [0.84, 0.94] | < 0.001 |
|  |  |  | Researcher 3 | |  |  |
|  | 363/365  (0.99) | 1233/1279  (0.96) | 363/408  (0.89) | 1233/1233 (1) | 0.92 [0.88, 0.97] | < 0.001 |

**Table S2** Agreement (%) between researcher (R1-R3) gonotrophic cycle classifications and the AR dataset for both *An. arabiensis* and *An. funestus*

| ***An. arabiensis*** |  | | | | |
| --- | --- | --- | --- | --- | --- |
| **Blood-feeding** | **R1 (%)** | **R2 (%)** | **R3 (%)** | **Mean (%)** | **SD** |
| 0 BF | 100 | 100 | 100 | 100 | 0.0 |
| 1 BF | 84 | 86 | 86 | 86 | 1.1 |
| 2 BF | 88 | 88 | 97 | 91 | 5.5 |
| 3 BF | 74 | 76 | 76 | 75 | 1.0 |
| 4 BF | 45 | 46 | 44 | 45 | 0.8 |
| ***An. funestus*** |  |  |  |  |  |
| 0 BF | 100 | 100 | 100 | 100 | 0.0 |
| 1 BF | 79 | 86 | 88 | 84 | 5.1 |
| 2 BF | 86 | 94 | 97 | 93 | 5.6 |
| 3 BF | 69 | 69 | 65 | 68 | 2.2 |
| 4 BF | 33 | 36 | 35 | 35 | 1.3 |

**Table S3.** Multinomial-Dirichlet overlap scores for *An. arabiensis*

0 = no overlap with AR | 1 = perfect match |

| **Blood Feeding** | **Comparison** | **Score** | **95% CI** |
| --- | --- | --- | --- |
| BF0 | AR vs R1 | 1.00 | [0.99-0.99] |
| BF0 | AR vs R2 | 1.00 | [0.99-0.99] |
| BF0 | AR vs R3 | 1.00 | [0.99-0.99] |
| BF1 | AR vs R1 | 0.84 | [0.80-0.89] |
| BF1 | AR vs R2 | 0.86 | [0.82-0.90] |
| BF1 | AR vs R3 | 0.86 | [0.82-0.90] |
| BF2 | AR vs R1 | 0.88 | [0.83-0.92] |
| BF2 | AR vs R2 | 0.88 | [0.83-0.92] |
| BF2 | AR vs R3 | 0.97 | [0.94-0.99] |
| BF3 | AR vs R1 | 0.75 | [0.69-0.79] |
| BF3 | AR vs R2 | 0.76 | [0.71-0.81] |
| BF3 | AR vs R3 | 0.76 | [0.71-0.81] |
| BF4 | AR vs R1 | 0.46 | [0.43-0.48] |
| BF4 | AR vs R2 | 0.46 | [0.43–0.49] |
| BF4 | AR vs R3 | 0.45 | [0.42-0.48] |

Excellent (≥0.90) | Good/Moderate (0.70-0.89) | Poor (<0.70)

**Table S4.** Multinomial-Dirichlet overlap scores for *An. funestus*

0 = no overlap with AR | 1 = perfect match |

| **Blood Feeding** | **Comparison** | **Score** | **95% CI** |
| --- | --- | --- | --- |
| BF0 | AR vs R1 | 1.00 | [0.99-0.99] |
| BF0 | AR vs R2 | 1.00 | [0.99-0.99] |
| BF0 | AR vs R3 | 1.00 | [0.99-0.99] |
| BF1 | AR vs R1 | 0.79 | [0.73-0.83] |
| BF1 | AR vs R2 | 0.86 | [0.81-0.90] |
| BF1 | AR vs R3 | 0.88 | [0.84-0.92] |
| BF2 | AR vs R1 | 0.87 | [0.81-0.92] |
| BF2 | AR vs R2 | 0.95 | [0.89-0.99] |
| BF2 | AR vs R3 | 0.97 | [0.92-0.99] |
| BF3 | AR vs R1 | 0.69 | [0.64-0.74] |
| BF3 | AR vs R2 | 0.69 | [0.64-0.75] |
| BF3 | AR vs R3 | 0.66 | [0.65-0.71] |
| BF4 | AR vs R1 | 0.34 | [0.32-0.36] |
| BF4 | AR vs R2 | 0.36 | [0.33-0.39] |
| BF4 | AR vs R3 | 0.36 | [0.33-0.39] |

Excellent (≥0.90) | Good/Moderate (0.70-0.89) | Poor (<0.70)

**Table S5.** Inter-rater reliability of parity classification (nulliparous vs parous) by three researchers for *An. arabiensis* and *An. funestus*

## Species Researcher Researcher Cohen's IRR *Kappa*(κ)

**P-value**

Nulliparous n/N (prop)

Parous n/N (prop)

Nulliparous n/N (prop)

Parous n/N (prop)

## [95 % CI]

| **Researcher 1 Researcher 2** | | | | | | |
| --- | --- | --- | --- | --- | --- | --- |
| 446/531 | | 1396/1477 | 446/518 | 1396/1490 | 0.80 [0.76, 0.84] | <0.001 |
| (0.84) | | (0.95) | (0.86) | (0.94) |  |  |
| **Researcher 1 Researcher 3** | | | | | | |
| ***An. arabiensis*** | 445/531 | 1401/1477 | 445/520 | 1401/1499 | 0.80 [0.77, 0.84] | <0.001 |
|  | (0.84) | (0.95) | (0.86) | (0.93) |  |  |
| **Researcher 2 Researcher 3** | | | | | | |
|  | 444/518 | 1415/1490 | 444/520 | 1415/1499 | 0.82 [0.78, 0.85] | <0.001 |
|  | (0.86) | (0.95) | (0.85) | (0.94) |  |  |
| **Researcher 1 Researcher 2** | | | | | | |
| ***An. funestus*** | 380/466 | 1179/1244 (0.95) | 380/445 | 1179/1265 | 0.80 [0.76, 0.84] | < 0.001 |
|  | (0.82) |  | (0.85) | (0.93) |  |  |
| **Researcher 1 Researcher 3** | | | | | | |
| 387/466 | | 1196/1244 | 387/435 | 1196/1275 | 0.83 [0.79, 0.87] | < 0.001 |
| (0.83) | | (0.96) | (0.89) | (0.94) |  |  |
| **Researcher 2 Researcher 3** | | | | | | |
|  | 395/445 | 1225/1265 | 395/435 | 1225/1275 | 0.88 [0.84, 0.92] | < 0.001 |
|  | (0.89) | (0.97) | (0.91) | (0.96) |  |  |

**Table S6.** Pairwise inter-rater agreement in gonotrophic cycle classification (GC0-GC3) between researchers for *Anopheles arabiensis* and *Anopheles funestus*.

| **Species** | **Researcher** | | | | **Researcher** | | | | **Cohen’s IRR**  ***Kappa*(κ)**  **[95 % CI]** | **P-value** |
| --- | --- | --- | --- | --- | --- | --- | --- | --- | --- | --- |
|  | **GC0**  **n/N (prop)** | **GC1**  **n/N (prop)** | **GC2**  **n/N (prop)** | **GC3**  **n/N (prop)** | **GC0**  **n/N (prop)** | **GC1**  **n/N (prop)** | **GC2**  **n/N (prop)** | **GC3**  **n/N (prop)** |  |  |
|  | **Researcher 1** | | | | **Researcher 2** | | | |  |  |
|  | 446/531 | 286/440 | 316/528 | 394/516 | 446/518 | 286/456 | 316/521 | 394/520 | 0.58 [0.56, | <0.001 |
|  | (0.84) | (0.65) | (0.60) | (0.76) | (0.86) | (0.63) | (0.61) | (0.76) | 0.61] |  |
| ***Anophele*** |  |  |  |  |  |  |  |  |  |  |
| ***s*** | **Researcher 1** | | | | **Researcher 3** | | | |  |  |
| ***arabiensis*** | 445/531 | 265/440 | 354/528 | 393/516 | 445/520 | 265/408 | 354/579 | 393/508 | 0.60 [0.57, | <0.001 |
|  | (0.84) | (0.60) | (0.67) | (0.76) | (0.86) | (0.65) | (0.61) | (0.77) | 0.63] |  |
|  | **Researcher 2** | | | | **Researcher 3** | | | |  |  |
|  | 444/518 | 269/456 | 338/521 | 391/520 | 444/520 | 269/408 | 338/579 | 391/508 | 0.59 [0.57, | <0.001 |
|  | (0.86) | (0.59) | (0.65) | (0.75) | (0.85) | (0.66) | (0.58) | (0.77) | 0.63] |  |
|  |  |  |  |  |  |  |  |  |  |  |
|  | **Researcher 1** | | | | **Researcher 2** | | | |  |  |
|  | 380/465 | 209/356 | 286/479 | 279/406 | 380/443 | 209/362 | 286/498 | 279/403 | 0.55 [0.52, | < 0.001 |
|  | (0.82) | (0.59) | (0.60) | (0.69) | (0.86) | (0.58) | (0.57) | (0.69) | 0.57] |  |
|  | **Researcher 1** | | | | **Researcher 3** | | | |  |  |
|  | 387/466 | 224/358 | 296/479 | 272/405 | 387/435 | 224/366 | 296/517 | 272/390 | 0.57 [0.54, | < 0.001 |
| ***Anophele*** | (0.83) | (0.63) | (0.62) | (0.67) | (0.89) | (0.61) | (0.57) | (0.7) | 0.59] |  |
| ***s funestus*** |  |  |  |  |  |  |  |  |  |  |
|  | **Researcher 2** | | | | **Researcher 3** | | | |  |  |
|  | 393/443 | 249/363 | 302/497 | 261/403 | 393/432 | 249/367 | 302/517 | 261/390 | 0.58 [0.56, | < 0.001 |
|  | (0.89) | (0.69) | (0.61) | (0.65) | (0.91) | (0.68) | (0.58) | (0.67) | 0.61] |  |

**Table S7.** Inter-rater reliability for parity classification (Field-collected mosquitoes)

| **Species** | **Overall (Fleiss’ κ)** | **Pairwise Comparison** | **Cohen’s κ** | **95% CI** | **p-value** |
| --- | --- | --- | --- | --- | --- |
| *An. arabiensis*  (n=559) | **0.87** | R1 vs R2 | 0.933 | [0.89-0.97] | <0.001 |
|  |  | R1 vs R3 | 0.845 | [0.79-0.89] | <0.001 |
|  |  | R1 vs R4 | 0.865 | [0.81-0.92] | <0.001 |
|  |  | R2 vs R3 | 0.880 | [0.83-0.93] | <0.001 |
|  |  | R2 vs R4 | 0.901 | [0.86-0.95] | <0.001 |
|  |  | R3 vs R4 | 0.822 | [0.76-0.88] | <0.001 |
| *An. funestus*  (n=507) | **0.86** | R1 vs R2 | 0.927 | [0.89-0.97] | <0.001 |
|  |  | R1 vs R3 | 0.821 | [0.76-0.88] | <0.001 |
|  |  | R1 vs R4 | 0.863 | [0.81-0.92] | <0.001 |
|  |  | R2 vs R3 | 0.859 | [0.81-0.91] | <0.001 |
|  |  | R2 vs R4 | 0.912 | [0.87-0.95] | <0.001 |
|  |  | R3 vs R4 | 0.785 | [0.72-0.85] | <0.001 |

**Table S8.** Inter-rater reliability for GC classification (Field-collected mosquitoes)

| **Species** | **Overall (Fleiss’**  **κ)** | **Pairwise**  **Comparison** | **Cohen’s κ** | **95% CI** | **p-value** |
| --- | --- | --- | --- | --- | --- |
| *An. arabiensis*  *(n=521)* | **0.88** | R1 vs R2 | 0.93 | [0.89-0.97] | <0.001 |
|  |  | R1 vs R3 | 0.85 | [0.79-0.90] | <0.001 |
|  |  | R1 vs R4 | 0.88 | [0.83-0.93] | <0.001 |
|  |  | R2 vs R3 | 0.88 | [0.83-0.93] | <0.001 |
|  |  | R2 vs R4 | 0.91 | [0.87-0.96] | <0.001 |
|  |  | R3 vs R4 | 0.83 | [0.77-0.89] | <0.001 |
| *An. funestus* | **0.87** | R1 vs R2 | 0.93 | [0.89-0.97] | <0.001 |
| (n=459) |  |  |  |  |  |
|  |  | R1 vs R3 | 0.83 | [0.77-0.87] | <0.001 |
|  |  | R1 vs R4 | 0.88 | [0.83-0.93] | <0.001 |
|  |  | R2 vs R3 | 0.86 | [0.80-0.91] | <0.001 |
|  |  | R2 vs R4 | 0.92 | [0.88-0.97] | <0.001 |
|  |  | R3 vs R4 | 0.79 | [0.73-0.86] | <0.001 |
